# Body Randomization Reduces the Sim-to-Real Gap for Compliant Quadruped Locomotion

**DOI:** 10.3389/fnbot.2019.00009

**Published:** 2019-03-28

**Authors:** Alexander Vandesompele, Gabriel Urbain, Hossain Mahmud, Francis wyffels, Joni Dambre

**Affiliations:** ^1^AIRO, Electronics and Information Systems Department, Ghent University-Imec, Ghent, Belgium; ^2^fortiss GmbH, Munich, Germany

**Keywords:** compliant robotics, quadruped control, knowledge transfer, simulation-reality gap, dynamics randomization

## Abstract

Designing controllers for compliant, underactuated robots is challenging and usually requires a learning procedure. Learning robotic control in simulated environments can speed up the process whilst lowering risk of physical damage. Since perfect simulations are unfeasible, several techniques are used to improve transfer to the real world. Here, we investigate the impact of randomizing body parameters during learning of CPG controllers in simulation. The controllers are evaluated on our physical quadruped robot. We find that body randomization in simulation increases chances of finding gaits that function well on the real robot.

## 1. Introduction

Compliant robots can provide many benefits over rigid robots (Pfeifer and Iida, 2007). They are more versatile and posses an inherently greater capacity to deal with different environments or with changing body properties due to wear and tear. Additionally, they can be more energy-efficient, safer for humans and less costly. The drawback is that they are generally more difficult to control than rigid robots.

Currently, state-of-the-art robots are usually made of rigid components (e.g., Raibert et al., 2008; Barasuol, 2013; Park et al., 2017). The rigid and well characterized body parts allow for controllers to be explicitly designed, based on accurate knowledge of the robot's physical properties. There are, however, some severe limitations to this approach. It is prohibitively difficult to design controllers that can adapt to a wide variety of environments and to the changing body properties due to wear and tear over the robot's lifetime. Well characterized and reliable components also come at a high cost.

The same approach cannot be applied to compliant robots, as their body parts can interact highly non-linearly with each other and the robots environment. This makes it difficult to accurately model their physical properties. Machine learning approaches are promising to the development of adaptive controllers for compliant robots. The combination of machine learning and compliant robotics may lead to robots moving out of highly standardized environments and into daily life at a cost that is affordable for consumers.

In the field of robot locomotion, machine learning techniques have been increasingly successful in developing adaptive robot controllers in simulation. Especially in the field of deep reinforcement learning, there have been some significant improvements recently (Heess et al., 2017; Peng et al., 2017). These controllers are usually learned in simulation and not on the physical robot. Learning only on the robot is challenging for multiple reasons, it is usually time-costly and unoptimized controllers may damage the robot. While it is impossible to simulate the real world, it is desirable to optimize controllers as far as possible before training on the physical robot. Particularly, in the case of a locomotion controller, it is desirable to start on the physical robot with a stable gait to prevent damage.

### 1.1. Related Work

The transfer of knowledge obtained in one domain to a new domain is important to speed up learning. Knowledge transfer can be applied across tasks, where knowledge from a learned task is utilized to speed up learning a new task by the same model (Hamer et al., 2013; Um et al., 2014). For instance, transfer of a quadruped gait learned in a specific environment, speeds up learning in other environments (Degrave et al., 2015). Knowledge transfer can also be applied across models, for instance if knowledge obtained by a first robot is utilized by a second robot (Gupta et al., 2017) or if a model is trained in simulation and then applied to a physical robot (Peng et al., 2018). However, the transfer of knowledge from simulation to reality has proven challenging for locomotion controllers due to discrepancies between simulation and reality, the so-called *simulation-reality gap* (Lipson and Pollack, 2000). This gap can easily cause a controller that is optimized in simulation to fail in the real world. Different methods have been developed to decrease the gap, they can generally be divided into two categories: (i) improving simulation accuracy and (ii) improving controller robustness.

*System identification* improves simulation accuracy by tuning the simulation parameters to match the behavior of the physical system. In the *embodiment theory* framework (Füchslin et al., 2013), the relation between environment, body and controller is described from a dynamical view point, where each entity can be modeled as a non-linear filter. Improving the simulator accuracy is then reduced to matching the transfer function of these filters. Urbain et al. (2018) provides an automated and parametrized calibration method that improves simulation accuracy by treating both the physical robot and its parametrized model as black box dynamical systems. It optimizes the similarity between the transfer functions by matching their sensor response to a given actuation input.

Similarly, simulation accuracy can be improved with machine learning techniques. For instance, in computer vision tasks (e.g., Taigman et al., 2016; Bousmalis et al., 2017) and visually guided robotic grasping tasks (Bousmalis et al., 2018), synthetic data has been augmented with generative adversarial networks (GANs). The augmentation improves the realism of the synthetic data and hence results in better models.

Another approach for minimizing the simulation-reality gap is by increasing robustness of the learned controllers. This can be achieved by perturbing the simulated robot during learning or by adding noise to the simulated environment (*domain randomization*, Jakobi, 1998; Tobin et al., 2017). The assumption is that if the model is trained on a sufficiently broad range of simulated environments, the real world will seem like just another variation to the model. Similarly, *dynamics randomization* is achieved by randomizing physical properties. Tan et al. (2018) found that dynamics randomization decreased performance but increased stability of a non-compliant quadruped robot. In Mordatch et al. (2015), optimization on ensembles of models instead of only the nominal model enables functional gaits on a small humanoid. In Peng et al. (2018), dynamics randomization was necessary for sim-to-real transfer of a robotic arm controller.

### 1.2. Our Approach

Whereas, Tan et al. (2018) observed the benefit of dynamics randomization for quadrupedal gait stability, the platform used is a stable, commercial robot used in a non-compliant manner. Passive compliance and underactuation are considered important for robots to cope with a broad range of real-world environments (Pfeifer et al., 2012; Laschi and Cianchetti, 2014). However, the difficulty of modeling the robot accurately increases with compliance and underactuation as well as with the use of low-cost components, exacerbating the simulation-reality gap. In this work we investigate the impact of dynamics randomization on controller robustness for compliant quadruped locomotion.

Measuring the robots physical properties does not necessarily translate into a good model. Especially with compliant robots, the dynamics of the model may be different from the physical robot. Therefor, we use a calibration method that focuses on replicating the dynamics, as described in a previous paper Urbain et al. (2018).

Using the calibrated model, we investigate if and how body randomization reduces the simulation-reality gap. For this purpose, we restrain ourselves to a straightforward controller optimization: a parametrized *central pattern generator* (CPG) optimized with an evolutionary strategy (the *CMA-ES* algorithm). The optimization is repeated for varying degrees of body randomization and subsequently tested on the physical robot. The randomization is applied to body parameters critical for the robot dynamics: mass distribution, spring stiffness and foot friction.

We observed that randomization of body parameters on average improves the stability of gaits when applied to the physical robot. Additionally, the used method is relatively straightforward to implement.

## 2. Materials and Methods

### 2.1. Robot

**Figure 1:**
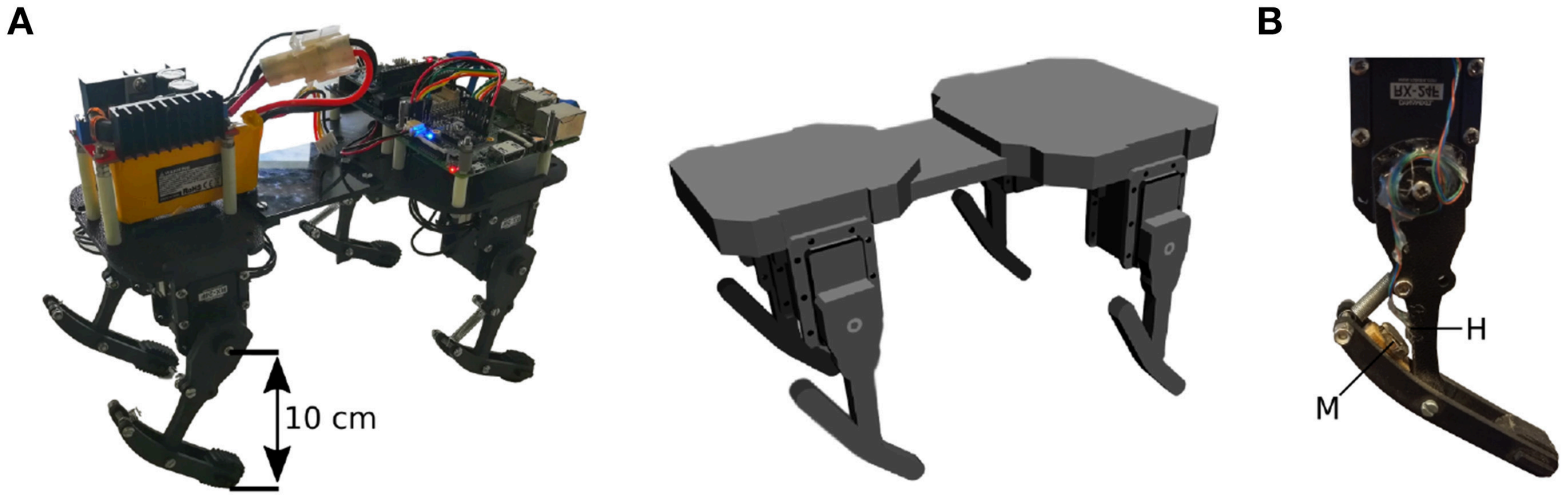
**(A)** The Tigrillo robot used in this paper (left) and its parametrized model in Gazebo (right). **(B)** Zoom on a leg with a spring loaded on the knee joint. M denotes the magnet, H denotes the Hall sensor.

The robot used for this paper is an update of the Tigrillo robot (Willems et al., 2017) as described by Urbain et al. (2018) (Figure 1A). Tigrillo is a low-cost platform built with off-the-shelf components and a structure laser cut out of ABS. It is developed for researching compliance in quadrupeds and has underactuated legs. Each hip joint is actuated with a Dynamixel RX-24F servomotor. The knee joints are passive compliant due to mounted springs (Figure 1B), which can be replaced to tune the passive compliance properties. The angle of the passive joints is measured with Hall sensors and rare-earth magnets placed on respectively the upper and lower leg parts. The Hall sensor will output a voltage between 0 and 5 V proportionally to the magnetic field. As the sensed magnetic field varies non-linearly with the distance to the magnet, the sensor provides us with non-linear body feedback. The total weight is 950 g and the robot fits in a box of 30 × 18 cm. The front legs are 15 cm apart and the hind legs 11 cm. A mounted *Raspberry Pi 3* allows wireless control of the robot from a remote computer. Actuator and sensor communication runs on the *Robotic Operating System (ROS*^1^*)*.

### 2.2. Calibration

The goal of the calibration process is to tune a simulated model to increase similarity in dynamics of the model and robot. The Tigrillo platform has a parametric model (Figure 1) that is simulated in the *Neurorobotics platform* (NRP) (Falotico et al., 2017), using Gazebo configured with ODE (Drumwright et al., 2010) physics engine. The model is calibrated using the calibration method detailed by Urbain et al. (2018). This method is an automated procedure in which both the model and real robot are considered sensor-to-actuator transfer functions. As the model is parametrized, its transfer function can be adapted by tuning the parameters.

We start with learning the sensor-to-actuator transfer function from the physical robot by recording the Hall sensor activity in response to an actuation pattern *a*(*t*). The actuation pattern is chosen to be a succession of sine waves at three different frequencies (0.4, 0.8, and 1.6 Hz). In order to calibrate the model such that it behaves similarly to the real robot during actual gaits, the sine waves are also used in anti-phase between the front and hind legs, creating bounding-like movement. Hence, in total six actuation patterns are used in the calibration procedure. To reduce sensor noise, an average (N = 5) of multiple recordings is used as the target signal *y*. Figure 3 shows the actuation and corresponding sensor signals for the legs of the physical robot. The high frequency event in the actuation signal for the front legs at the transition from high to low frequency (15th s) is an artifact caused by the signal generator. It does not significantly impact the calibration procedure as it is an event of short duration.

**Figure 2:**
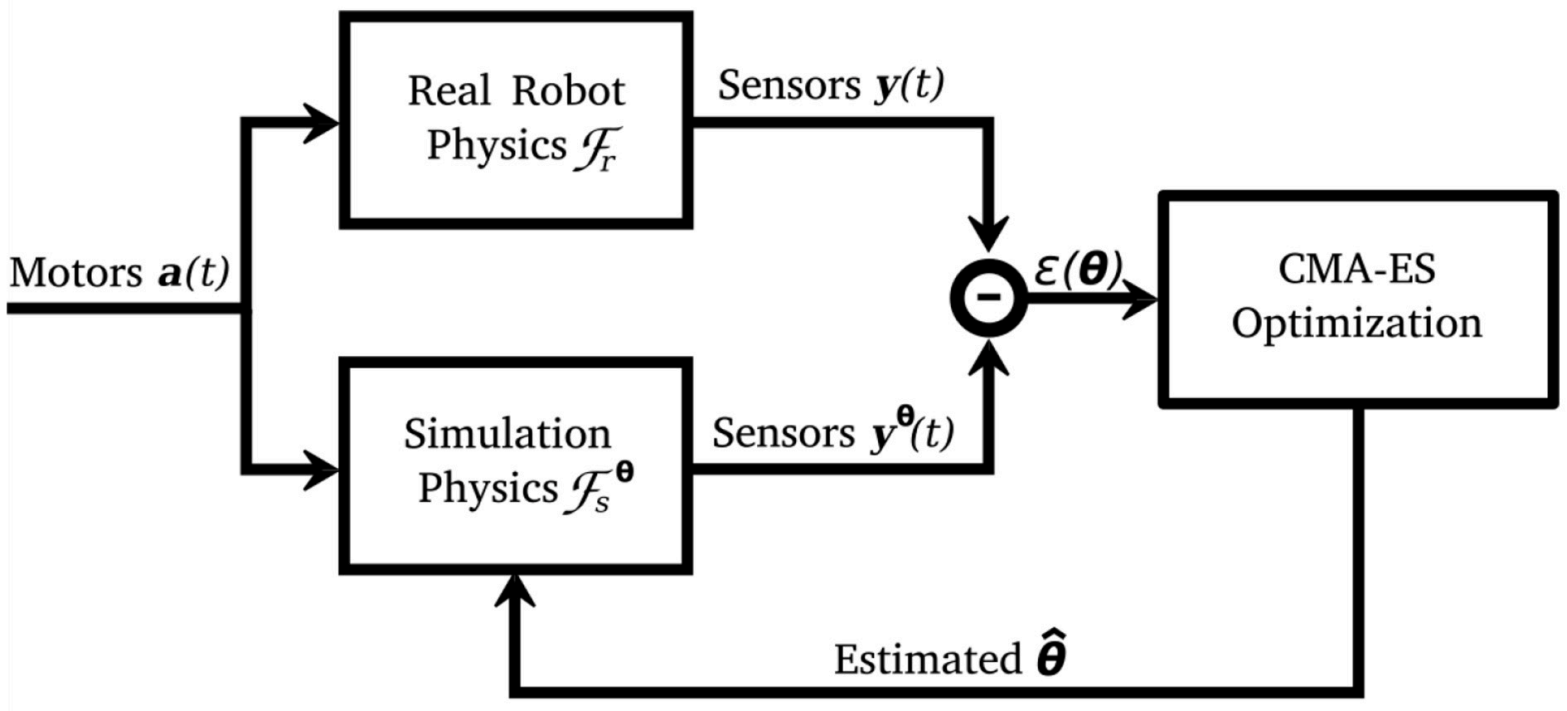
Diagram of the calibration procedure. CMA-ES optimization minimizes the difference between the sensor recording from the robot and the model (*y*(*t*) and *y*^*θ*^(*t*), respectively), by tuning the model parameters ($\hat{\theta }$). Figure adapted from Urbain et al. (2018).

Next, we want to tune the body parameters of the model to achieve a similar sensor-to-actuator transfer function. We start with an uncalibrated model based on the measured physical properties (see diagram in Figure 2). Then, *covariance matrix adaptation evolutionary strategy (CMA-ES)* is applied for the parameter search. The included parameters *θ* are those observed critical for the dynamic behavior and are listed in Table 1. The indices *f* and *h* refer to the front and hind part of the body, respectively. Parameter *θ*_*m*_ is the mass of the main body part on the front and hind side, *θ*_*μ*_ is the friction coefficient of the feet, and *θ*_*k*_ the spring constant indicating spring stiffness. The contact depth *θ*_*d*_ is the minimum depth before a contact correction impulse is applied. Parameter *θ*_*c*_ is the compression tolerance, which allows for the minimum angle of the passive joint to be smaller than the spring length, simulating spring compression.

**Figure 3:**
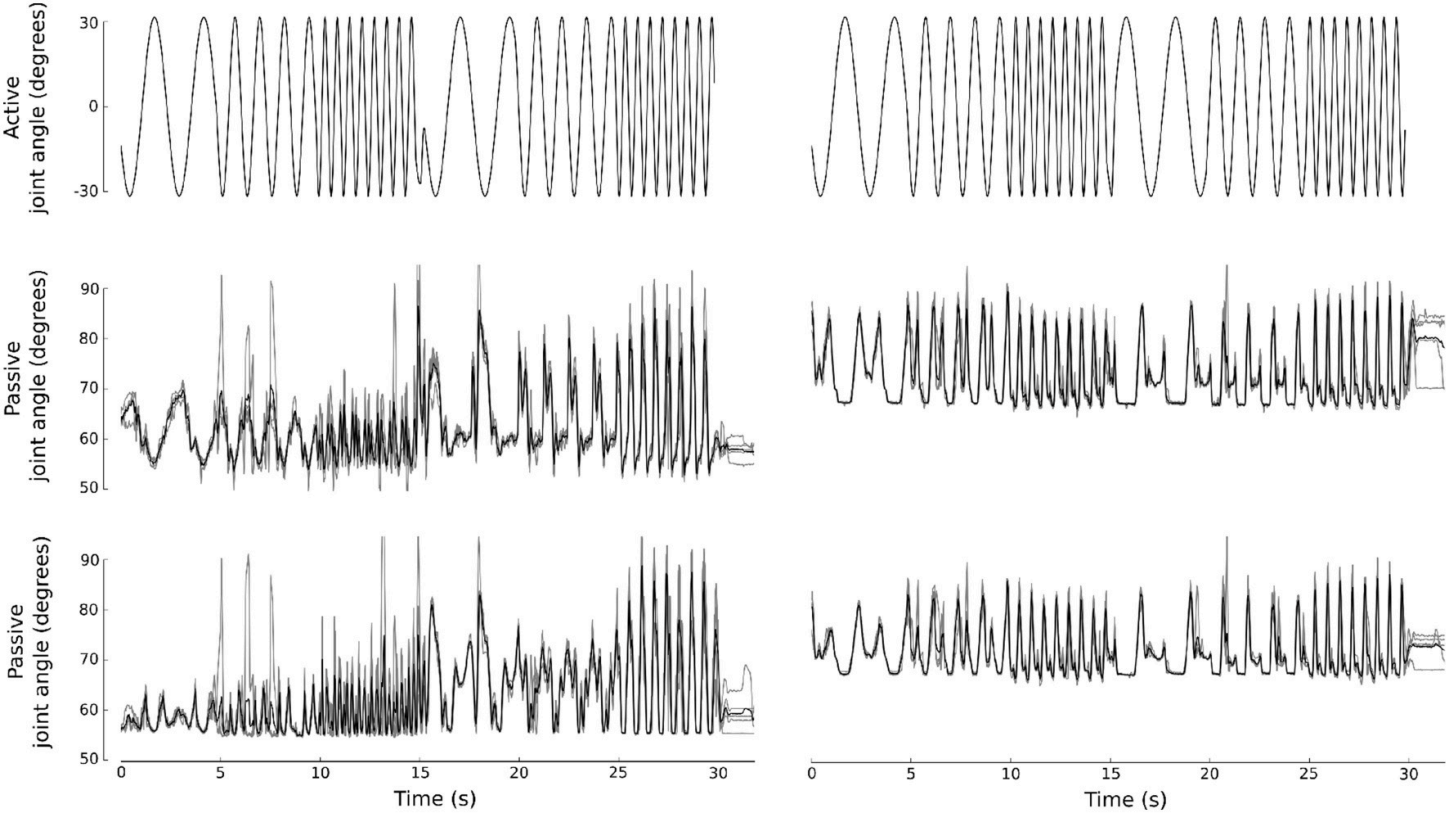
Characterization of the robot dynamics. The robot was actuated with a pattern of sine waves **(top row)**. The front legs **(left)** and hind legs **(right)** were actuated in phase firstly and in antiphase subsequently. The bottom rows show the Hall sensor readout in response to the actuation pattern for the front and hind legs (**left and right**, respectively). An average of 5 trials was used as target signal during model calibration.

**Table 1:** Parameters included in the calibration procedure with *CMA-ES*.

**Parameter**	**Description**	**Range**	**Unit**
*θ*_*mf*_	Front mass	[0.1, 0.5]	kg
*θ*_*mh*_	Hind mass	[0.1, 0.5]	kg
*θ*_μ*f*_	Front feet friction coefficient	[10^−3^, 2.]	*NA*
*θ*_μ*h*_	Hind feet friction coefficient	[10^−3^, 2.]	*NA*
*θ*_*df*_	Front feet contact depth	[10^−4^, 10^−2^]	m
*θ*_*dh*_	Hind feet contact depth	[10^−4^, 10^−2^]	m
*θ*_*kf*_	Front legs spring constant	[50, 10^3^]	N/m
*θ*_*kh*_	Hind legs spring constant	[50, 10^3^]	N/m
*θ*_*cf*_	Front compression tolerance	[0.92, 0.98]	mm
*θ*_*ch*_	Hind compression tolerance	[0.7, 1.2]	mm

A more detailed description of the *CMA-ES* algorithm can be found in Hansen (2006). It is an evolutionary algorithm that samples solutions from a multi-variate normal distribution. Every iteration, the mean and the covariance matrix of the distribution are updated. The mean is updated to increase the likelihood of previously successful solutions. The covariance matrix is updated to increase the likelihood of a previously successful search step. *CMA-ES* is well suited for a search space with multiple local minima. It requires few initial parameters and doesn't require derivation of the search space.

*CMA-ES* will minimize the error *ε*(*θ*), here chosen to be the root mean square error (*RMSE*) with *y* being the target sensor signal as recorded on the robot and ŷ the sensor signal recorded in simulation:

(1) $$\begin{array}{r} \hat{\theta }=\underset{\theta }{\mathrm{argmin}}\varepsilon \left(\theta \right) \end{array}$$

(2) $$\varepsilon (\theta )=\sqrt{\frac{\Sigma_{i=1}^{n}{\left(\hat{y}-y\right)}^{2}}{n}}$$

### 2.3. Gait Search

#### 2.3.1. Central Pattern Generator

With the calibrated model, a controller is optimized in the same simulation environment. The controller is modeled by a parametrized CPG, based on the open-loop CPG introduced by Gay et al. (2013). The CPG is described by three equations:

(3) $$\begin{array}{l} \dot{r}=\gamma (\mu -r^{2})r\\ \dot{\phi }=\omega \\ \lambda =r\text{cos}(\phi_{L})+o, \end{array}$$

Where *r* describes the radius of the oscillator and *ϕ* the current phase. Both are used to calculate the actual control value *λ* in degrees. *μ* is the target amplitude of the oscillator and γ is a positive gain that defines the convergence speed of the radius to the target amplitude. ω is the radial frequency of the oscillator and *o* the offset. *ϕ*_*L*_ is a filter applied on the phase of the oscillator, the formula of which is different for the swing and stance phase of the control as determined by the duty factor (*d*):

(4) $$\phi_{L}=\{\begin{array}{ll} \frac{\phi_{2\pi }}{2d} & \text{if }\phi_{2\pi }<2\pi d\\ \frac{\phi_{2\pi }+2\pi (1-2d)}{2(1-d)} & \text{otherwise} \end{array}$$

$$\text{and }\phi_{2\pi }=\phi \;(\text{mod }2\pi )$$

The Tigrillo platform has four actuated joints that are controlled by four phase-coupled CPGs. One leg, the front left, is chosen as reference leg and three phase offset (*po*) parameters describe the phase difference of the remaining 3 legs to the reference leg. This is implemented by adding a term to the formula for the phase (*ϕ*) in Equation (3). For instance, for the coupling between the front left and front right oscillators:

(5) $$\begin{array}{rc} \dot{\phi_{fr}} & =\omega +w_{fr}sin\left(\phi_{fl}-\phi_{fr}-po_{fr}\right) \end{array}$$

where *w*_*fr*_ is the coupling strength.

#### 2.3.2. Gait Search With CMA-ES

The CMA-ES algorithm is used again to optimize the CPG controller. The search space consist of a subset of the CPG parameters. To enforce a walking gait, the search space is constrained to the set of parameters as detailed in Table 2. The walking gait is characterized by a phase offset among the legs that results in asymmetry along the transverse axis. Additionally, the Tigrillo robot has no feet retraction mechanism in its underactuated legs. Consequently, maintaining balance during a walking-like gait presents a challenge for this platform. The frequency ω is fixed at 2π radian/s (1 Hz).

**Table 2:** Parameters and their ranges included in the *CMA-ES* optimization for a walking gait.

**Parameter**	**Symbol**	**Range**	**Unit**
Front amplitude	*μ*_*f*_	[45, 140]	degrees
Hind amplitude	*μ*_*h*_	[45, 140]	degrees
Front duty cycle	*d*_*f*_	[0.15, 0.85]	*NA*
Hind duty cycle	*d*_*h*_	[0.15, 0.85]	*NA*
Front offset	*o*_*f*_	[-45, 10]	degrees
Hind offset	*o*_*h*_	[-45, -10]	degrees
Front right phase offset	*po*_*fr*_	[165, 195]	degrees
Hind left phase offset	*po*_*hl*_	[255, 285]	degrees
Hind right phase offset	*po*_*hr*_	[75, 105]	degrees

*CMA-ES* as described by Hansen (2006) was used to perform the optimization, but with a larger population size (*N* = 20) to increase chance of avoiding local optima. Each solution is evaluated for 10 s of simulation time. As score function the distance of the model from origin after 10 s is used. After convergence of the *CMA-ES* algorithm, the best performing individual of the final generation is chosen as the final solution. Hence each optimization resulted in one set of CPG parameters that corresponds to a gait.

**Figure 4:**
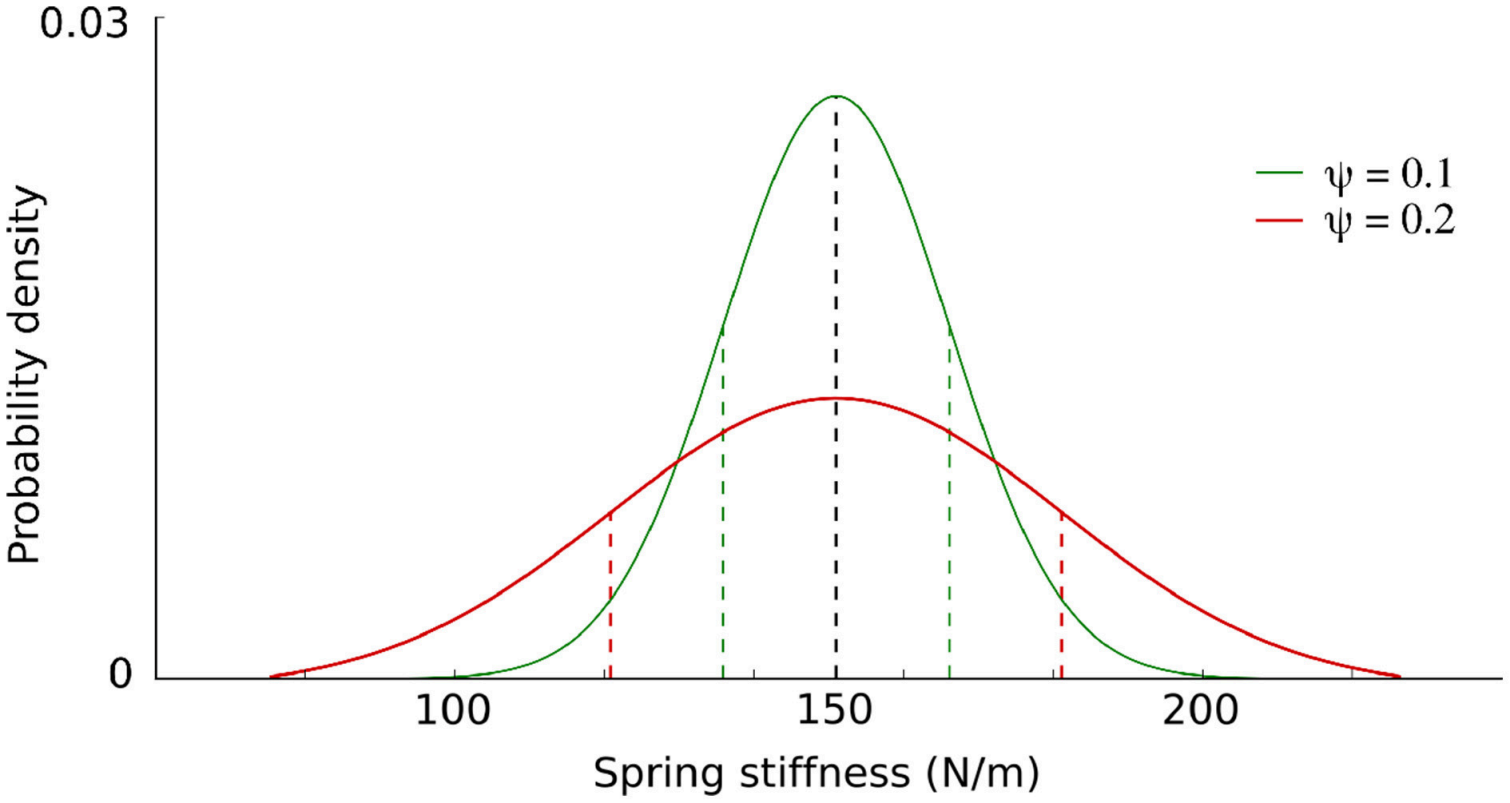
Randomization level *ψ* affects the sampling distribution of the body parameters. *ψ* determines the standard deviation (σ, colored dashed lines) of the Gaussian distribution with mean *μ* (black dashed line). In this example for parameter *θ*_*k*_, σ = *ψ* ^*^ *μ*, with *μ* = 151*N*/*m* being the spring stiffness of the calibrated model front legs.

To investigate the effect of randomizing body morphology on transferability, *CMA-ES* optimizations were performed with varying levels of randomization of body properties deemed critical for the gait dynamics: *θ*_*k*_, *θ*_*μ*_, and *θ*_*m*_. The parameters are sampled from a Gaussian distribution with the mean value *μ* taken from the calibrated model and the randomization parameter *ψ* affecting the standard deviation σ of the Gaussian distribution in a parameter dependent fashion (see Figure 4 for an example). Given *ψ*, the standard deviation is obtained by the following equations, for the parameters *θ*_*k*_ and *θ*_*m*_:

(6) $$\begin{array}{r} \sigma =\psi \mu \end{array}$$

And, for the parameters *θ*_*μ*_:

(7) $$\begin{array}{r} \sigma =2\psi \mu \end{array}$$

For *θ*_*m*_, the mass of the main front body part is sampled from the Gaussian distribution and the mass of the main hind body part is adapted such that the total mass remains constant, varying only the mass distribution. *θ*_*k*_ and *θ*_*μ*_ are sampled independently per leg, hence each individual has 9 variable parameters. Because the noisy body parameters are sampled from a distribution, it is desirable to evaluate a given controller on multiple independent trials. It was observed that the average score over 5 trials gave a reliable estimate.

### 2.4. Evaluation Methods

In all experiments performance and stability are measured. Stability is measured as the fraction of all trials in which the model or robot has fallen. In simulation, performance is measured as distance between the original and final position of the model after a short time period (10 s unless mentioned otherwise). For the physical robot, the robot is tracked with a Kinect camera and performance is measured as distance traveled by the robot after a short time period (10 s unless mentioned otherwise).

## 3. Results

### 3.1. Calibration

**Figure 5:**
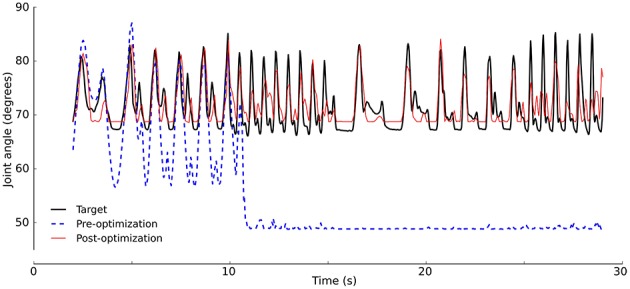
Model calibration. The model was optimized to match the robot sensor response (“target”, black). Sensor values after calibration (red, RMSE= 0.245) match better than before calibration (blue, RMSE = 0.741). Signals shown are for the hind right leg.

The aim of the calibration is to tune the robot model to achieve a sensor response to an actuation signal that is similar to that of the physical robot. Figure 5 shows the response of the model pre- and post-calibration. The calibration resulted in a model that approximates the dynamics of the physical robot. In line with the hypothesis of body randomization, we do not deem it beneficial to fine tune calibration to the greatest extent possible. Even with an optimally calibrated model, the simulation-reality gap may remain. Rather, we try to bridge the gap by searching for a gait that works on a variety of body morphologies. The calibrated model serves as a default morphology, on which variations are applied.

### 3.2. Gait Optimization in Simulation

To evaluate the effect of body randomization on the simulation-reality gap, gait optimizations with different levels of randomization were performed (parameter *ψ* ranging from 0 to 0.4). A higher level of randomization means that the body parameters were sampled from a broader distribution. Since the *CMA-ES* optimization does not guarantee an optimal convergence, experiments were repeated 5 times.

**Figure 6:**
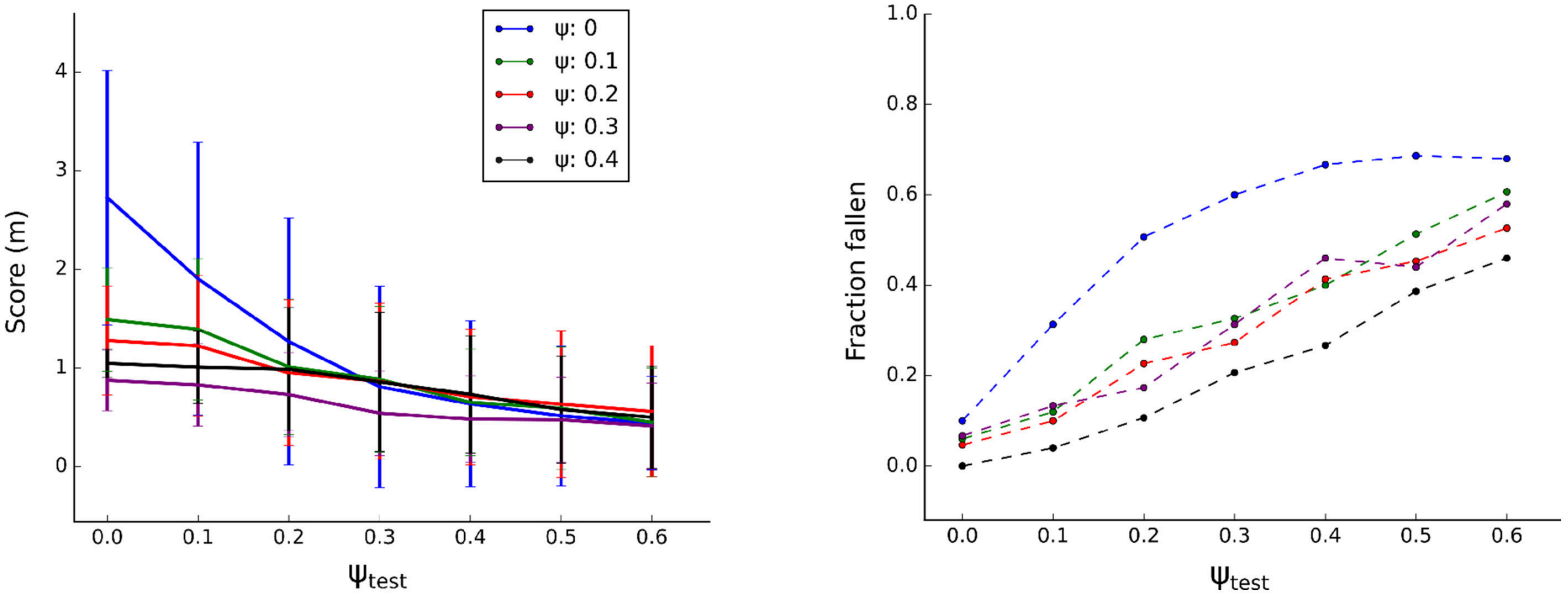
Gait evaluation in simulation. For each level of *ψ*, 5 optimizations were performed resulting in 5 controllers. Each controller was tested in 30 trials. **Left:** average distance to origin (in 20 s, *N* = 150). **Right:** observed frequency of falling over, normalized.

For each optimization, the solution was chosen as best performing individual of the final generation. Subsequently, the performance of each solution was tested in simulation. The controller, trained with a specific level of *ψ*, was tested on varying degrees of randomization (*ψ*_*test*_). For each level of *ψ*, the procedure was repeated 5 times, Figure 6, Left presents the average performance. Performance of solutions trained on the nominal body (without body randomization, *ψ* = 0) is higher if tested on bodies with no or limited randomization (*ψ*_*test*_ < 0.3) and converges with other solutions in the higher randomization regimes (*ψ*_*test*_ ≥ 0.3). The variance of these solutions however is higher, reflecting the performance variation both between solutions and between trials of the same solution. Solutions trained with randomization (*ψ* ≥ 0.1) have a lower score when tested without randomization (*ψ*_*test*_ = 0), because they have developed more prudent locomotion during training as the randomization prevents overfitting of the controller to the dynamics of the simulation environment and model.

Figure 6, Right plots the robustness metric: frequency of falling over. As expected, the fraction of trials resulting in a robot fallen over increases with increasing body randomization (*ψ*_*test*_). More importantly, the amount of randomization during training improves stability of the resulting solution. The gaits trained without randomization (*ψ* = 0) are particularly susceptible to losing balance when tested on body configurations that it is not trained on. Overall, it seems there is a trade-off between speed and stability of a given solution. Randomization impacts this trade-off and favors more prudent gaits that are slower but more stable.

**Figure 7:**
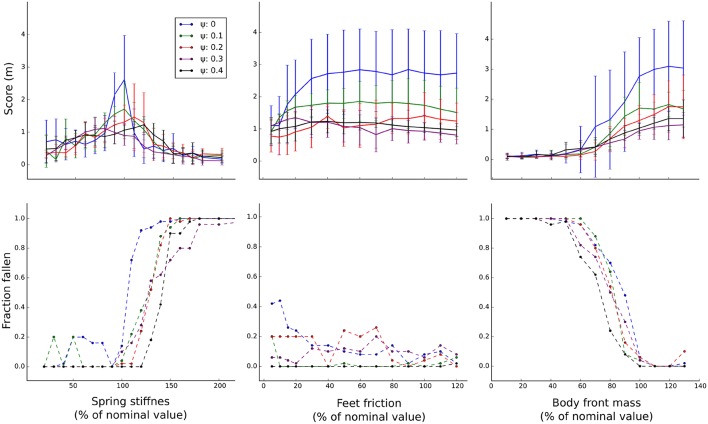
Evaluation of parameters. Gaits are evaluated while incrementally varying a single parameter at a time, other parameters are kept at the default value. **Top:** average performance measured as distance from origin after 20 s (*N* = 50). **Bottom:** observed frequency of falling over, normalized.

To evaluate the impact of variation of the different body parameters, the gaits were also tested while incrementally varying a single body parameter at the time and keeping other parameters at their default value (Figure 7). Similar to the previous test, training with body randomization lowers average performance but also the variance when changing the feet friction and mass distribution parameters. Varying the spring stiffness parameter has a more dramatic effect on the performance and here body randomization seems to improve performance in certain parameter ranges. Generally, the negative impact of varying body parameters on stability is reduced by increasing the amount of training randomization (Figure 7, Bottom).

### 3.3. Transfer to Real World

**Figure 8:**
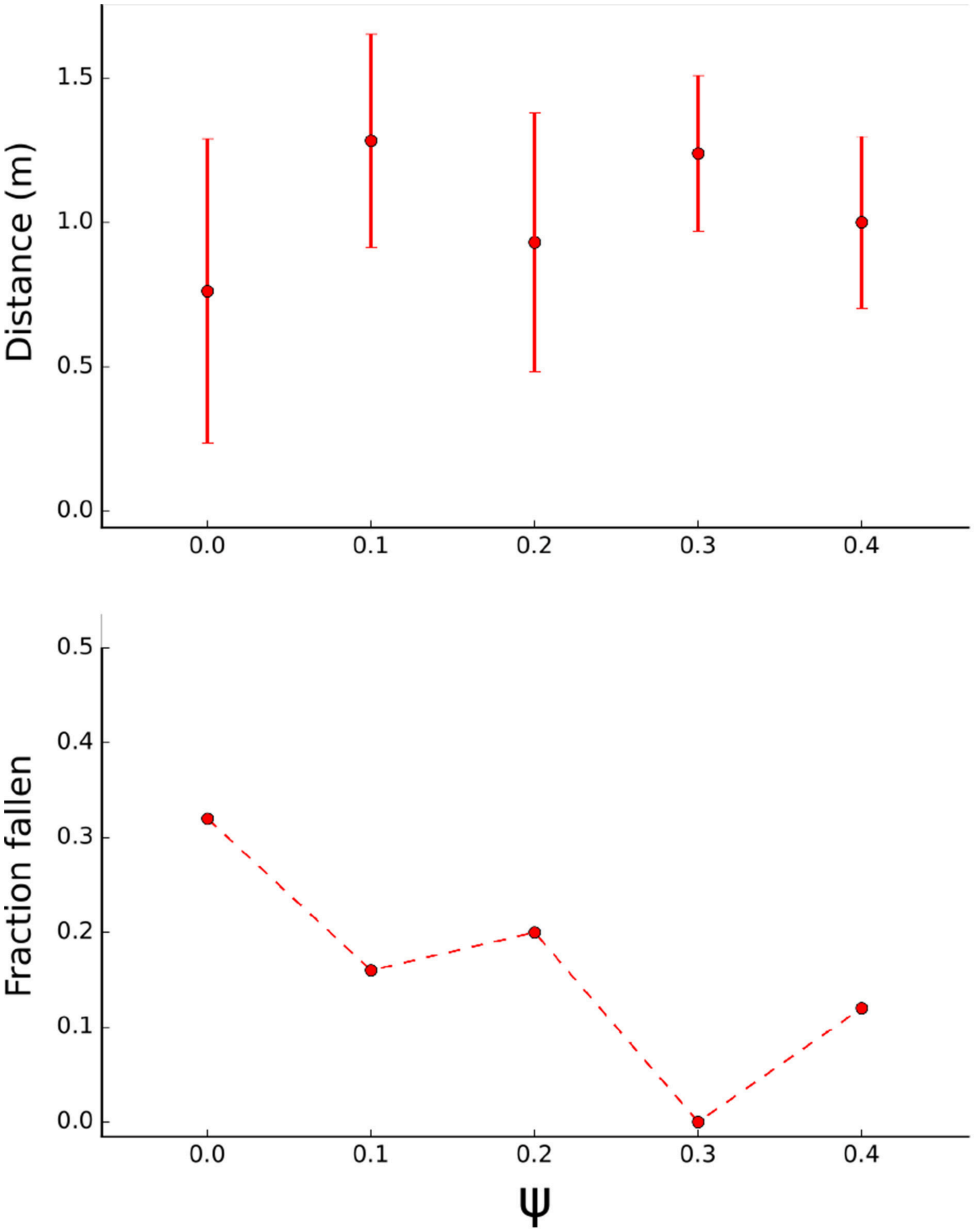
Transfer test on physical robot. **Top:** Average traveled distance of the robot in 10 s trials. Each point represents an average of *N* = 25 trials (each result of the optimizations was tested 5 times on the robot), error bars indicate standard deviation. **Bottom:** fraction of trials where the robot flipped to its side or back.

The final solution of each optimization was tested on the physical robot. Performance is measured as distance traveled in 10 s (Figure 8, Top). Generally, adding body randomization (*ψ* > 0) improves average performance and decreases the variability in performance. Forty percent (2/5) of optimizations without randomization (*ψ* = 0) resulted in a functional gait compared to 80% (16/20) of optimizations with randomization (*ψ* > 0). Non-functional gaits result in the robot shuffling in place or consistently falling within 10 s. While a randomization level *ψ* > 0 seems beneficial, the precise level of randomization doesn't seem critical. This could be a consequence of sampling the parameters from a Gaussian distribution around a common mean. The optimization procedure was repeated with a very high randomization level (*ψ* = 2, not shown), which resulted in nonfunctional gaits. Presumably, the gaits learned without randomization (*ψ* = 0) are overfit to the training environment and hence perform well on the nominal body in the simulation, but suffer a performance drop when tested in another setting such as on the physical robot.

Additionally, frequency of falling was recorded (Figure 8, Bottom). Lack of body randomization resulted in a higher probability of the robot falling to its side or back. Optimizations with body randomization generally resulted in reduced frequency of falling, using *ψ* = 0.3 resulted in functional gaits that maintained balance in all trials.

## 4. Conclusion

In this work, we investigated bridging the simulation-reality gap for a compliant, underactuated robot, by treating a robot and its model as variations of the same dynamical system. Consequently, both the calibration and control optimization procedure focus on body parameters critical for the behavior of the dynamical system.

For the optimization procedure, we showed that body randomization results in improved transferability of the controllers. Lack of randomization results in better performance in simulation but worse performance on the real robot, compared to the optimization with randomization. Addition of randomization also improved stability of controllers, both in simulation and on the physical robot. Body randomization can be interpreted as a regularization method, preventing the optimization procedure from overfitting to the particular simulation environment. While body randomization improves sim-to-real transfer, the precise amount of randomization did not seem critical. For our platform, the use of body randomization enhances the quality of controllers learned in simulation. The resulting controller has an improved stability, reducing risk of physical harm and providing a safe starting point to continue learning on the physical platform. This method is relatively straightforward to implement and could be used in combination with other tools that reduce the simulation-reality gap, such as domain randomization and data augmentation.

From the evaluations of gaits in simulation, it is clear that the quality of a given gait can be very sensitive to even small changes in physical properties such as the stiffness of springs in the leg. It would therefor be interesting to use a platform with adaptive spring stiffness in future work. This would allow to tune the compliance in function of gait optimization.

## Data Availability

The datasets generated for this study are available on request to the corresponding author.

## Author Contributions

The experiments were conceived by AV, GU, Fw, and JD and designed by AV. The physical platform was co-developed by GU, the virtual platform by HM. The data were analyzed by AV with help of Fw and JD. The manuscript was written by AV, with comments and corrections from GU, HM, Fw, and JD.

### Conflict of Interest Statement

The authors declare that the research was conducted in the absence of any commercial or financial relationships that could be construed as a potential conflict of interest.

## Footnotes

^1^http://wiki.ros.org/
